# Cost-effectiveness of a hybrid emergency room system for severe trauma: a health technology assessment from the perspective of the third-party payer in Japan

**DOI:** 10.1186/s13017-020-00344-x

**Published:** 2021-01-07

**Authors:** Takahiro Kinoshita, Kensuke Moriwaki, Nao Hanaki, Tetsuhisa Kitamura, Kazuma Yamakawa, Takashi Fukuda, Myriam G. M. Hunink, Satoshi Fujimi

**Affiliations:** 1 Division of Trauma and Surgical Critical Care, Osaka General Medical Center, 3-1-56 Bandai-Higashi, Sumiyoshi-ku, Osaka, 558-8558 Japan; 2 Master of Public Health Program, Harvard T.H. Chan School of Public Health, Boston, MA USA; 3 Comprehensive Unit for Health Economic Evidence Review and Decision Support (CHEERS), Research Organization of Science and Technology, Ristumeikan University, #209, Research Park Bid. No. 2, 134, Minami-machi, Chudoji, Simogyo-ku, Kyoto, 600-8813 Japan; 4 Department of Public Health, Graduate School of Medicine, Osaka University, 2-2, Yamada-oka, Suita, 565-0871 Japan; 5 Division of Environmental Medicine and Population Sciences, Department of Social and Environmental Medicine, Graduate School of Medicine, Osaka University, 2-2 Yamada-oka, Suita, 565-0871 Japan; 6 Center for Outcomes Research and Economic Evaluation for Health, National Institute of Public Health, 2-3-6 Minami, Wako, Saitama, 351-0197 Japan; 7 Department of Radiology, Erasmus MC University Medical Centre Rotterdam, Rotterdam, Netherlands; 8 Department of Epidemiology, Erasmus MC University Medical Centre Rotterdam, Rotterdam, Netherlands; 9 Centre for Health Decision Sciences, Harvard T H Chan School of Public Health, Boston, MA USA

**Keywords:** HERS, ICER, Markov model, QALY, Utility

## Abstract

**Background:**

Hybrid emergency room (ER) systems, consisting of an angiography-computed tomography (CT) machine in a trauma resuscitation room, are reported to be effective for reducing death from exsanguination in trauma patients. We aimed to investigate the cost-effectiveness of a hybrid ER system in severe trauma patients without severe traumatic brain injury (TBI).

**Methods:**

We conducted a cost-utility analysis comparing the hybrid ER system to the conventional ER system from the perspective of the third-party healthcare payer in Japan. A short-term decision tree and a long-term Markov model using a lifetime time horizon were constructed to estimate quality-adjusted life years (QALYs) and associated lifetime healthcare costs. Short-term mortality and healthcare costs were derived from medical records and claims data in a tertiary care hospital with a hybrid ER. Long-term mortality and utilities were extrapolated from the literature. The willingness-to-pay threshold was set at $47,619 per QALY gained and the discount rate was 2%. Deterministic and probabilistic sensitivity analyses were conducted.

**Results:**

The hybrid ER system was associated with a gain of 1.03 QALYs and an increment of $33,591 lifetime costs compared to the conventional ER system, resulting in an ICER of $32,522 per QALY gained. The ICER was lower than the willingness-to-pay threshold if the odds ratio of 28-day mortality was < 0.66. Probabilistic sensitivity analysis indicated that the hybrid ER system was cost-effective with a 79.3% probability.

**Conclusion:**

The present study suggested that the hybrid ER system is a likely cost-effective strategy for treating severe trauma patients without severe TBI.

## Introduction

**Fig. 1:**
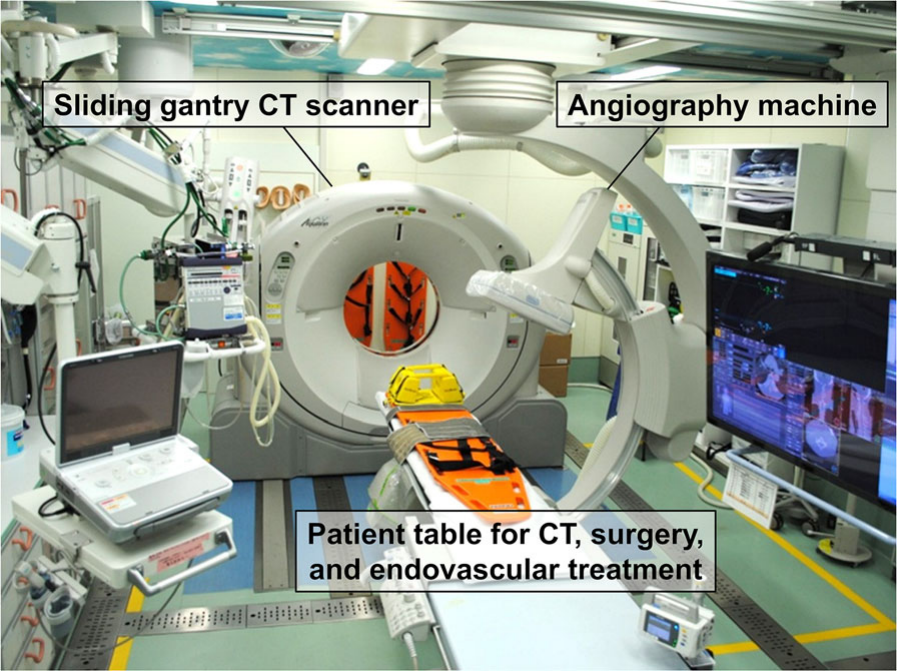
Picture of the angiography-CT equipment installed in the hybrid ER. CT scanning, REBOA, emergency surgery, and endovascular treatments are performed in this room without patient transfer. CT, computed tomography; ER, emergency room; REBOA, resuscitative endovascular balloon occlusion of the aorta

In the last decade, two radiological procedures, that is, computed tomography (CT) and angiography are of increasing importance for the management of severe trauma [1, 2]. Several trauma centers in Europe have reported that the installation of a CT scanner in trauma resuscitation rooms dramatically reduces the time to perform CT examinations and come to a definitive diagnosis [3, 4]. Moreover, endovascular treatments using angiography including resuscitative endovascular balloon occlusion of the aorta (REBOA) and angioembolization are considered to improve outcomes of trauma patients with appropriate indications [5, 6]. In 2011, we installed an angiography-CT in a trauma resuscitation room in order to obtain immediate access to both CT imaging and interventional procedures (Fig. 1). As this system enabled us to conduct all “examinations” and “treatments” without any patient transfer, we named it the “hybrid emergency room (ER).” We previously reported that a novel trauma workflow using a hybrid ER decreased time to start bleeding control procedures and significantly reduced deaths from exsanguination [7].

Although 11 trauma centers in Japan and 1 trauma center in South Korea already have installed the hybrid ER [8], most trauma centers in the world manage patients with the standard trauma workflow based on the Advanced Trauma Life Support (ATLS) guidelines without angiography-CT equipment. One of the hurdles for spreading the hybrid ER system is the valuable capital investments not only for the installation of the angiography-CT machine but also for the reconstruction of ER and the maintenance of the equipment. Although this system is considered to be effective for life-threatening hemorrhagic trauma patients, whether it is worth the costs for these investments remains uncertain [7]. Thus, cost-effectiveness analysis is required to support decisions for policy-makers and/or insurance payers to facilitate investments for a hybrid ER to improve health-related quality of life among populations for which they are responsible.

The aim of the present study was to investigate the cost-effectiveness of the hybrid ER using a framework of economic evaluation. We assessed whether the novel trauma workflow using a hybrid ER was cost-effective compared to the standard trauma workflow using a conventional ER in the Japanese healthcare setting.

## Methods

### Study design

We conducted a model-based cost-utility analysis from the perspective of the third-party healthcare payer in Japan. The intervention of interest was the novel trauma workflow using a hybrid ER and the comparator was the standard trauma workflow based on the ATLS guidelines without angiography-CT equipment. Outcomes analyzed were quality-adjusted life years (QALYs), costs, and the incremental cost-effectiveness ratio (ICER), which is the incremental cost associated with a new therapy needed to generate one additional QALY, since this methodology for economic evaluation is commonly used in health system payers and health technology assessment organizations [9, 10]. As the costs were recorded in Japanese yen (JPY), we converted them into US dollars (105 JPY = $1 USD). The willingness-to-pay for one additional QALY gained was set to $47,619, which is equivalent to 5 million JPY, the current threshold willingness-to-pay for a QALY in Japan [11]. The annual discount rate was set at 2% in both costs and utilities based on Japanese guidelines for economic evaluations of healthcare [12].

The study was approved by the Institutional Review Board of the Osaka General Medical Center (S201916010). The board waived the need for informed consent, as this was a modeling study based on retrospective data collection only.

### Study population

The modeled population was severe blunt trauma (injury severity score ≥ 16) patients without severe traumatic brain injury (TBI) (Glasgow coma scale ≤ 8 with intracranial hemorrhage demonstrated by CT). According to our previous study, cases of traumatic cardiopulmonary arrest on arrival, pediatric patients younger than 15 years of age, patients who were transferred to other hospitals within 24 h after admission, penetrating trauma patients, and pregnant women were excluded [7].

### Model structure

**Fig. 2:**
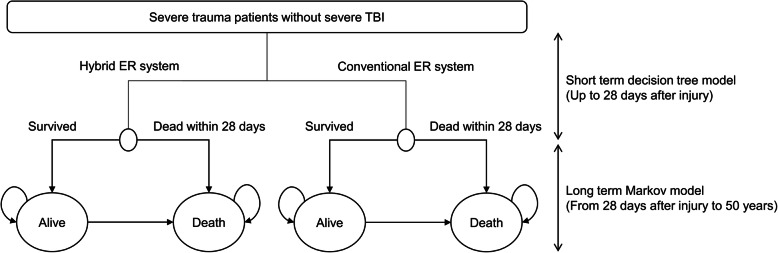
Model structure. Initial admission costs were included in the short-term decision tree and follow-up costs were included in the long-term Markov model. Capital investment costs were added on the admission costs only to the hybrid ER system. Twenty-eight-day mortality was used for the transition probability in the decision tree. Extrapolated 1- to 3-year mortality rates after trauma and Japanese life table were used for the transition probability in the Markov model. QALYs were calculated using utility of intensive care patients in the decision tree and long-term utility after severe trauma in the Markov model. The only differences between two strategies were initial transition probability, initial admission costs, and capital investment costs. ER, emergency room; QALY, quality-adjusted life year

Figure 2 shows model structure. We constructed a short-term decision tree and a long-term Markov model to determine the QALYs, life years (LYs), and costs associated with the conventional ER system and the hybrid ER system. All patients started with the “severe trauma” state and then transitioned to the “survived” state or “dead” state at 28 days after injury. Patients in the “survived” state either stayed in the “survived” state or moved to the “dead” state. The “dead” state was defined as the absorbing state. We set the initial age as 50 years according to the mean age of the previous study population [7]. The length of time for the first decision tree was defined as 28 days and the cycle length for the Markov model was set as 1 year. The model was run until either death or fiftieth year, assuming that no patients survived after the age of 100 years. The model was developed and analyzed using TreeAge Pro 2019 (TreeAge Software, Williamstown, MA, USA).

### Transition probabilities

The initial transition probabilities from the “severe trauma” state to the “dead” state, which were the 28-day mortalities in the two groups, were derived from our previous study cohort [7]. In the conventional group, 28-day mortality was directly calculated from the observed data. We conducted a multivariable logistic regression to estimate the odds ratio (OR) and its 95% confidence interval (CI) adjusting for clinically plausible or known confounders: heart rate, body temperature, hemoglobin, lactate, prothrombin time-international normalized ratio, and probability of survival using Trauma and Injury Severity Score. The 28-day mortality in the hybrid ER group (*P*_1_) was obtained from the 28-day mortality in the conventional group (P_0_) and OR as follows:

$$ {Odds}_0=\frac{P_0}{1-{P}_0},{Odds}_1={Odds}_0\times OR,{P}_1=\frac{Odds_1}{1+{Odds}_1} $$

For the transition probabilities from the “survived” state to the “dead” state, the same probabilities were used in both groups. Mortalities at first, second, and third year were extrapolated from an observational study that reported the 1- and 3-year mortalities of major adult blunt trauma survivors [13]. A transition probability (*P*) of death occurring over a time interval (*t*) with hazard rate (*r*) was calculated according to the following formula [14].

$$ P=1-{e}^{- rt} $$

The transition probabilities in the fourth year and later were based on Japanese life tables and calculated with a weighted average of males and females in the general population using the proportion of male and female patients in the cohort [15].

### Costs

Healthcare-related costs for the initial admission in each group were obtained from the claims data in our hospital. We categorized them into surgical costs, transfusion costs, and hospitalization costs including pharmaceutical and procedural costs. These costs were analyzed in the short-term decision tree. We also investigated annual follow-up costs of the patients that survived. First to fifth year follow-up costs were directly obtained from the claims data. For the sixth year and later, we used the same costs as for the fifth year.

Costs for installation of the hybrid ER were provided by a manufacturer (Canon Medical Systems Corp., Tochigi, Japan). The costs consisted of two different parts: the price of the equipment and initial reconstruction costs of the ER. As the angiography-CT machine is a long-lasting resource, a depreciation period of 6 years was chosen as the life of the investment. The amortized yearly expenditure for the angiography-CT machine (*M*) was calculated from the initial price of the machine (Pr) of $1,714,000, the annual interest rate of 1% (*i*), and the depreciation period of 6 years (*N*) using the following formula :[16]

$$ M=\mathit{\Pr}\times i\times \frac{{\left(1+i\right)}^{N-1}}{{\left(1+i\right)}^N-1} $$

In addition to the amortized cost, we included annual maintenance costs. The residual value of the angiography-CT machine was subtracted from the expenditure during the year following the depreciation period. We assumed that these costs were used to treat severe trauma patients who were transferred to the hybrid ER. As 270 patients were treated during a 4-year study period in the hybrid ER group, the discounted capital investment costs were divided by 405 patients (the estimated number of patients in the 6-year depreciation period) and added to the admission costs of patients in the hybrid ER group.

We did not include time costs, productivity costs, and other non-healthcare costs in the analysis.

### Utilities

To calculate QALYs, we extrapolated utility values from the literature [17, 18]. The utility in the first 28-day hospitalization period was derived from a study that assessed quality of life of critical care patients using the six-dimensional short-form health state questionnaire (SF-6D) [17]. Another study that used the EuroQol–Five Dimensions questionnaire (EQ-5D) for the health state assessment was selected to determine the utility in the follow-up period [18].

### Sensitivity analyses

We performed deterministic sensitivity analyses to assess the impact of various key parameters. The ranges for each parameter were determined by 95% CIs derived from the cohort data or publications if available. Otherwise, the plausible range was decided based on expert opinion. In addition to the costs explained above, we assumed that additional labor costs needed to operate the hybrid ER could be covered by third-party payers. We expected that annual cost to hire an additional physician was $150,000 per year. Similar to the investment costs, the discounted labor costs were divided by the annual number of patients and included only in the hybrid ER group.

Moreover, we conducted a probabilistic sensitivity analysis using second order Monte Carlo simulations to explore uncertainty in the input parameters. Values of parameters were randomly selected from the distribution of the input parameters and the model was run, which was repeated for 1000 simulations. We plotted the results on the cost-effectiveness plane and described the cost-effectiveness acceptability curve to estimate the proportion of simulations that the novel trauma workflow using hybrid ER would be preferred in terms of cost-effectiveness as function of the willingness-to-pay threshold.

## Results

### Costs, effects, and utilities

Cost, effects, utilities, and the discount rate were obtained from our previous study cohort or retrieved from other studies (Table S1, Supporting information). Compared to the conventional group, the admission cost was significantly higher in the hybrid ER group (conventional vs. hybrid ER; $60,742 [95% CI, $52,595 to 68,889] vs. $86,716 [95% CI, $76,388 to 97,044]; *p* < 0.0001). The 28-day mortality in the conventional group was 0.16 (95% CI, 0.12–0.20). The hybrid ER group had significantly lower odds of 28-day mortality compared to the conventional group (OR, 0.48; 95% CI, 0.24 to 0.92). Utility was calculated to be 0.57 in the severe trauma state and 0.70 in the survival state.

### Base case analysis

In the base-case scenario, the trauma workflow using hybrid ER was associated with an additional 1.03 QALYs and 2.05 LYs in the lifetime time horizon when compared to the standard trauma workflow using conventional ER. However, the hybrid ER system resulted in an increment of $33,591 in lifetime costs compared to the conventional ER system. As a result, the ICER of the trauma workflow using the hybrid ER was $32,522 per QALY gain (Table 1).

**Table 1:** Model-predicted cost-effectiveness from base case analysis

	Lifetime healthcare costs ($)	Lifetime QALYs	Life years	Difference in costs ($)	Difference in QALYs	ICER ($ per QALY gained)
Conventional ER	71,146	12.16	24.15	N/A	N/A	N/A
Hybrid ER	104,737	13.19	26.20	33,591	1.03	32,522

Lifetime healthcare costs were calculated from initial admission costs and follow-up costs using the short-term decision tree model and the long-term Markov model. Capital investment costs were only included in the hybrid ER. Lifetime QALYs were also measured using these models. ICER was defined as the incremental costs divided by the additional QALYs. *ER* emergency room, *ICER* incremental cost-effectiveness ratio, *QALY* quality-adjusted life year

### Deterministic sensitivity analysis

**Fig. 3:**
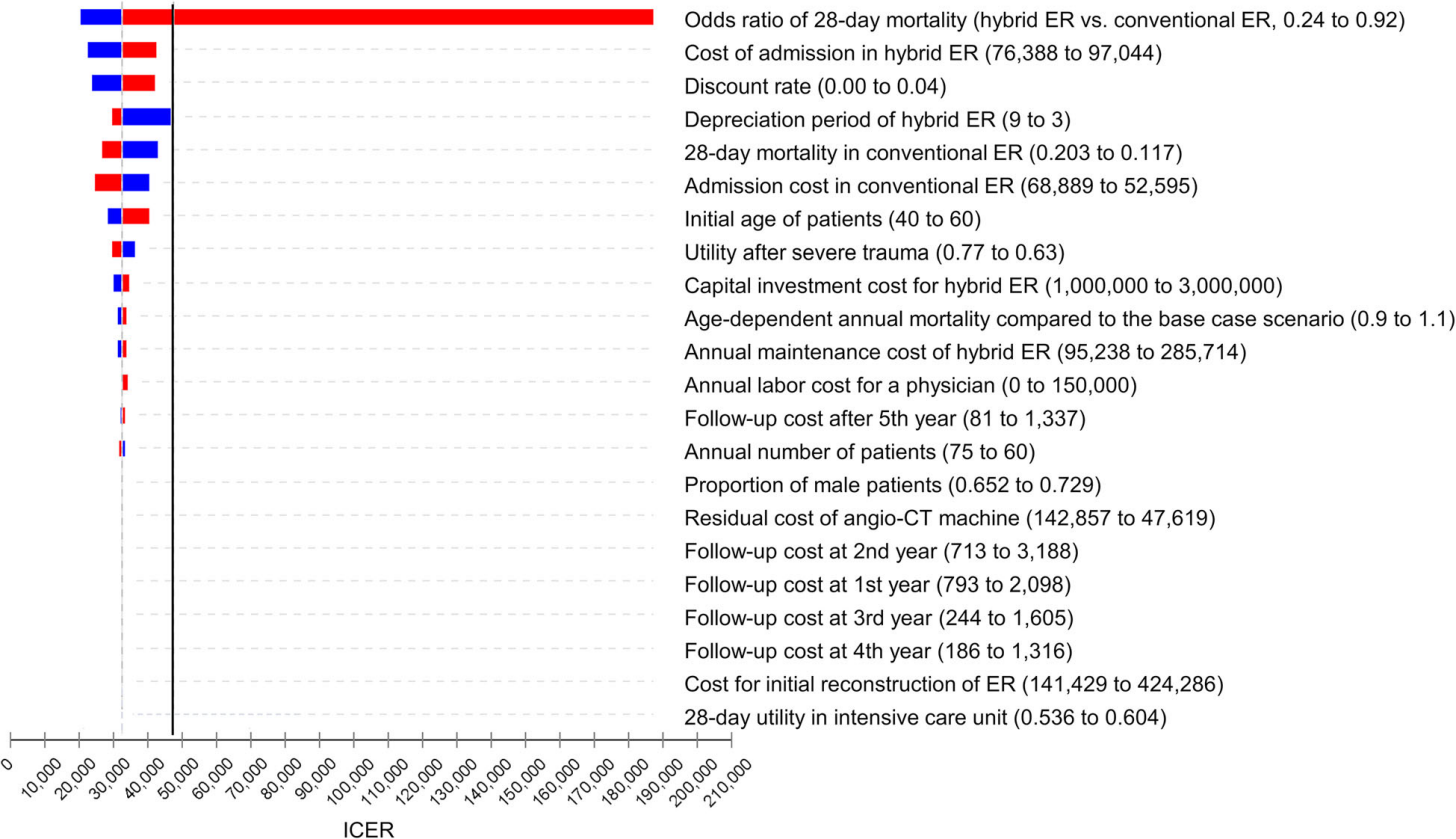
Deterministic sensitivity analysis. The tornado diagram showing the results of worst and best case scenario based on the plausible ranges of the parameters. The vertical solid line represents the willingness-to-pay threshold ($47,619), and the vertical dotted line represents the ICER from the base-case scenario ($32,522). Horizontal bars represent the estimated ICERs based on the plausible range of the parameters. ER, emergency room; ICER, incremental cost-effectiveness ratio

The results of the one-way deterministic sensitivity analysis are summarized in Fig. 3 using a tornado diagram. The ICER was most sensitive to the short-term treatment effect of the hybrid ER; the ICER varied from $ 20,358 per QALY to $ 209,042 per QALY according to the 95% CI of the OR of 28-day mortality. The calculated ICER became lower than the willingness-to-pay threshold of $47,619 (5 million JPY) when the OR of 28-day mortality was lower than 0.66. For all other parameters including the investment costs and the labor costs, the ICERs fell under the willingness-to-pay threshold even in the worst scenarios.

### Probabilistic sensitivity analysis

**Fig. 4:**
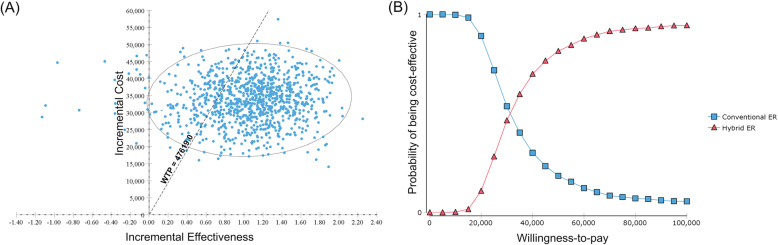
Probabilistic sensitivity analysis. **a** Cost-effectiveness scatterplot. Each plot shows a result of one simulation. Plots located in the first quadrants suggest that both cost and effect are higher in the hybrid ER system (trade-off) and plots located in the second quadrants suggest that cost is higher but effect is lower in the hybrid ER system (inferior). Plots reside under the dotted line of willingness-to-pay threshold suggest that the results of the simulations are cost-effective. **b** Cost-effectiveness acceptability curve. The line with square markers shows the probability that the conventional ER is cost-effective and the line with triangle markers shows the probability that the hybrid ER is cost-effective at each willingness-to-pay threshold. ER, emergency room

Figure 4 shows the results of probabilistic sensitivity analysis. The cost-effectiveness scatterplot revealed that most of the points were located in the right-upper quadrant and only a few points resided in the left-upper quadrant, indicating that the novel trauma workflow using hybrid ER was always more costly and most of the times more effective than the status quo. Based on the cost-effectiveness acceptability curves, the probability that the hybrid ER system became cost-effective was estimated to be 79.3% at the willingness-to-pay threshold of $47,619/QALY.

## Discussions

In the present study, we investigated whether effects of the hybrid ER system on lifetime QALYs justified valuable investment costs from the perspective of the third-party healthcare payer in Japan. The main finding was that the novel trauma workflow using hybrid ER was a likely cost-effective strategy compared to the standard trauma workflow using conventional ER; the ICER of the hybrid ER system was lower than the willingness-to-pay threshold according to the base case analysis. Moreover, the one-way deterministic sensitivity analysis revealed that the capital investment cost for hybrid ER had only small impact on the cost-effectiveness even though we assumed that overall $1 to 3 million was needed for the installation of the system. Similarly, adding physician’s annual compensation ($150,000 per year) in the hybrid ER group only marginally increased the ICER. These results suggested that the expenditure of health resources to this equipment was worthwhile. To the best of our knowledge, this was the first study that evaluated cost-effectiveness of the hybrid ER system, which installed an angiography-CT machine in a trauma resuscitation room to achieve immediate CT diagnosis and bleeding control procedures including REBOA, surgery, and angioembolization.

We conservatively selected the willingness-to-pay threshold of $47,619 (5 million JPY) for the analysis as the price of pharmaceutical or medical devices does not require any adjustment when the ICER falls under this lowest cut-off value in Japan [11]. Although the willingness-to-pay threshold is a matter of choice and open to discussion, it is still useful to calculate ICERs since it enables us to compare the cost-effectiveness of new treatments to other popular healthcare interventions. For example, Takura and colleagues have reported that the estimated ICER of maintenance hemodialysis is $68,800 ± 44,700 per QALY, suggesting that the hybrid ER system is a more cost-effective intervention than maintenance hemodialysis [19]. We believe that the willingness-to-pay threshold in this analysis is also acceptable in other developed countries as the World Health Organization’s suggestion of three times per capita gross domestic product ends up with the thresholds well over $100,000 per QALY in most high-income countries [20].

We limited the analysis to severe trauma patients without severe TBI since a previous study suggested that the significant effects of the hybrid ER on mortality were identified in exsanguinating patients [7]. As these rooms have been used also for severe TBI patients and non-trauma patients in several tertiary care hospitals [21–23], the estimated ICER obtained from the model excluding these patients could be imprecise. However, the hybrid ER system is expected to be cost-effective even if we include these patients in the analysis as the investment costs and the depreciation period will not change; the estimated ICER was already lower than the willingness-to-pay threshold under the conservative assumption that this room was annually used for only 68 severe trauma patients without severe TBI.

Generalizability of our results to other countries with different healthcare systems needs careful consideration. First, the costs for admission, follow-up, investments, and maintenance may be different across the countries. However, the results of one-way deterministic sensitivity analysis suggested that the ICER was not sensitive to the variation of these costs. Second, the initial age of 50 years should be modified based on different age distributions of trauma patients in other countries [24]. The deterministic sensitivity analysis also revealed that the result was robust to the starting age. Moreover, the estimated ICERs were always under the willingness-to-pay threshold even if we changed the values of utilities, which were known to be influenced by age, gender, socioeconomic status, and cultures of targeted populations [25–27]. The only parameter that led to an excess of the threshold was the OR of the 28-day mortality. Hence, whether severe trauma patients can benefit from the hybrid ER with the same mortality reduction rate is the critical issue when it comes to generalizing the results. We have previously pointed out that a multidisciplinary approach with appropriate assessment of CT, rapid and safe use of REBOA, and prompt and effective hemostasis including surgery and angioembolization is essential to maximize the benefit of the hybrid ER system [28]. Preparing these environments may also play a pivotal role in using this system wisely in terms of cost-effectiveness.

The present study has several limitations. First, the effect of the hybrid ER system was derived from neither randomized controlled trials nor systematic reviews. Although it is unlikely that this system worsens outcomes of trauma patients, the magnitude of beneficial effects should be confirmed in future studies. Second, given limited publications in this field, we extrapolated the utilities after trauma from studies conducted in other countries. Finally, the results might be biased as we excluded severe TBI patients and non-trauma patients who were treated in this room during the study period. However, the ICER would not have exceeded the willingness-to-pay threshold if we included these patients as the investment costs were constant.

## Conclusions

The novel trauma workflow using a hybrid ER, with both angiography and CT capabilities in a trauma resuscitation room, is a likely cost-effective strategy for treating severe trauma patients without severe TBI. The results of the present study would help healthcare decision-makers to judge whether it is worth to invest in the equipment to improve survival in target populations.

## Supplementary Information

**Additional file 1:.** Table S1. Input Parameters

## Data Availability

The datasets used and analyzed during the current study are available from the corresponding author on reasonable request.

## Abbreviations

ATLS: Advanced Trauma Life Support
CI: Confidence interval
CT: Computed tomography
EQ-5D: EuroQol–Five Dimensions questionnaire
ER: Emergency room
ICER: Incremental cost-effectiveness ratio
JPY: Japanese yen
LY: Life years
OR: Odds ratio
QALY: Quality-adjusted life year
REBOA: Resuscitative endovascular balloon occlusion of the aorta
SF-6D: Six-dimensional short-form health state questionnaire
TBI: Traumatic brain injury
USD: US dollars
