# Supplementary material for: Cost-effectiveness of a hybrid emergency room system for severe trauma: a health technology assessment from the perspective of the third-party payer in Japan
Source: World J Emerg Surg. 2021 Jan 7;16:2. doi: 10.1186/s13017-020-00344-x (PMC7791815; doi:10.1186/s13017-020-00344-x)
Supplement: Supplementary file 1 — Additional file 1:. Table S1. Input Parameters [file 13017_2020_344_MOESM1_ESM.docx]

**Table S1. Input Parameters**

| Parameter | Estimate | Range | Distribution | Reference |
| --- | --- | --- | --- | --- |
| Cost |  |  |  |  |
| Admission cost in conventional ER ($ per case) | 60,742 | 52,595 to 68,889 (95% CI) | Gamma | Original data |
| Surgical cost in conventional ER ($ per case) | 21,927 | 16,811 to 27,043 (95% CI) | – | Original data |
| Transfusion cost in conventional ER ($ per case) | 2,197 | 1,589 to 2,806 (95% CI) | – | Original data |
| Hospitalization cost in conventional ER ($ per case) | 36,618 | 32,380 to 40,856 (95% CI) | – | Original data |
| Admission cost in hybrid ER ($ per case) | 86,716 | 76,388 to 97,044 (95% CI) | Gamma | Original data |
| Surgical cost in hybrid ER ($ per case) | 36,052 | 28,968 to 43,135 (95% CI) | – | Original data |
| Transfusion cost in hybrid ER ($ per case) | 2,106 | 1,690 to 2,523 (95% CI) | – | Original data |
| Hospitalization cost in hybrid ER ($ per case) | 48,651 | 43,864 to 53,438 (95% CI) | – | Original data |
| Installation cost of hybrid ER ($) | 2,000,000 | ±50% | – | Assumption |
| Depreciation period of hybrid ER (year) | 6 | 3 to 9 | – | Assumption |
| Annual maintenance cost of hybrid ER ($) | 190,476 | ±50% | – | Assumption |
| Additional physicians cost ($) | 0 | 0 to 150,000 | – | Assumption |
| Total number of severe trauma patients without severe TBI | 270 | 240 to 300 | – | Original data |
| Case accumulation period of hybrid ER (year) | 4 | – | – | Original data |
| Follow up medical cost ($ in 1st year) | 1,379 | 793 to 2,098 (95% CI) | Gamma | Original data |
| Follow up medical cost ($ in 2nd year) | 1,769 | 713 to 3,188 (95% CI) | Gamma | Original data |
| Follow up medical cost ($ in 3rd year) | 770 | 244 to 1,605 (95% CI) | Gamma | Original data |
| Follow up medical cost ($ in 4th year) | 623 | 186 to 1,316 (95% CI) | Gamma | Original data |
| Follow up medical cost ($ after 5th year) | 500 | 81 to 1,337 (95% CI) | Gamma | Original data |
| Probability |  |  |  |  |
| Odds ratio of 28-day mortality (hybrid ER vs. conventional ER) | 0.48 | 0.24 to 0.92 (95% CI) | Log normal | Original data |
| 28-day mortality in conventional ER | 0.16 | 0.12 to 0.20 (95% CI) | Beta | Original data |
| 28-day mortality in hybrid ER | 0.084 | – | Conditional | N/A |
| Annual mortality in the first year | 0.075 | ±10% | Beta | [13] |
| Annual mortality in the second and third year | 0.050 |  | Conditional | [13] |
| Annual mortality in the fourth year and later | From life table |  | – | [15] |
| Patient age | 50 | 40 to 60 | – | Original data |
| Proportion of male patients | 0.691 | 0.652 to 0.729 (95% CI) | Beta | Original data |
| Utility |  |  |  |  |
| Utility in the intensive care unit (first 28 days) | 0.57 | 0.536 to 0.604 (95% CI) | Beta | [17] |
| Utility after severe trauma | 0.7 | ±10% | Triangle | [18] |
| Setting |  |  |  |  |
| Discount rate | 0.02 | 0 to 0.04 | – | N/A |

Admission costs, follow-up costs, short-term transition probability were obtained from medical records and claims data. Capital investment costs were provided by a manufacturer. First to third year transition probability and utilities were obtained from the literature. Fourth year and later probability was derived from the Japanese life table. ER, emergency room
